# The use of systems biology and immunological big data to guide vaccine development

**DOI:** 10.1186/s13073-015-0236-1

**Published:** 2015-11-10

**Authors:** Christoph J. Blohmke, Daniel O’Connor, Andrew J. Pollard

**Affiliations:** Oxford Vaccine Group, Centre for Clinical Vaccinology and Tropical Medicine, Department of Pediatrics, University of Oxford, The Churchill Hospital, Old Road, Oxford, OX3 7LE UK; NIHR Oxford Biomedical Research Center, The Churchill Hospital, Old Road, Oxford, OX3 7LE UK

## Abstract

High-throughput technologies applied to the analysis of vaccine responses are likely to reveal the mechanisms responsible for vaccine-induced protection, aid understanding of vaccine safety and help accelerate vaccine development and clinical trials.

## The need to understand vaccine responses

Vaccines are considered to be one of the most successful public health interventions in human history, with an estimated 20 million deaths averted between 2011 and 2020 [1] and significantly improved quality of life throughout the world. Understanding of the immunological processes underlying the ‘protection’ conferred by vaccines has been highly simplified as a result of the limitations of conventional immunological methodologies. Traditional vaccine development is based on empirical approaches, which are inherently time-consuming and costly. However, the recent development of multi-parameter cellular immunology, high-throughput proteomics and the advances in high-resolution genomics and transcriptomics now provide an opportunity to refine our understanding of the molecular mechanisms that underpin vaccine-conferred protection. Such in-depth knowledge will enable the rational design of vaccine candidates, reduce empiricism and lessen the burden of failed vaccine candidates, thereby expediting the development of successful vaccines.

Most vaccines have been designed to induce antibody (B-cell) responses to kill bacteria and neutralize viruses and toxins. Although antibodies are the primary mediators of the protection afforded by most vaccines, T-cell immunity (cell-mediated immunity) can also be detected following administration of routinely administered antigens and may be important in protection. For example, vaccination of individuals latently infected with varicella-zoster virus (VZV), the shingles vaccine, boosts VZV-specific T-cell immunity, which is believed to be the mechanism underlying subsequent prevention of viral reactivation.

Assessment of the magnitude of vaccine-specific immune responses (immunogenicity) and their immunological function is currently undertaken using a variety of assays (for example, the serum bactericidal assay or the plaque neutralization assay) and is usually linked to efficacy to permit licensure. Although standardized and reproducible, these assays only cover the end result (for example, antibody titers) of the complex interaction between a vaccine and the continuum of the innate and adaptive immune responses. A detailed understanding of the development of specific, functional and persistent immunity is missing, and this significantly hampers vaccine design.

## The power of systems biology

Acknowledging this limitation, recent high-throughput technological advances have revolutionized the way in which the immune system can be explored after vaccination and has prompted an increasingly pervasive doctrinal shift from reductionist to holistic or ‘systems biology’ research [2]. Systems biology aims to generate in-depth profiles by integrating multifaceted datasets and harnessing their power to predict, for example, immunogenicity following vaccination, using complex computational approaches. In particular, high-throughput methodologies for genomic and transcriptomic exploration have been at the forefront of this technological revolution; these were initially dominated by microarrays, but more recently microarrays have been superseded by next-generation sequencing (NGS).

Microarray technology was instrumental for the development of genome-wide association studies (GWASs), which have identified genetic variants associated with various human traits, including vaccine immunogenicity [3]. For example, a variant proximal to *HLA-DPB1* has been associated with responses to hepatitis B vaccine; intriguingly, this variant has also been associated with chronicity of hepatitis B infection, suggesting that a genetically determined inability to produce antibodies to hepatitis B surface antigen predisposes individuals to chronic hepatitis B infection [4]. Similarly, genetic variants associated with safety have been explored: variants in an interferon-stimulated gene (*IFI44L*) and the measles receptor (*CD46*) were found to be associated with febrile seizures following measles-mumps-rubella vaccination [5]. Understanding the genetic basis of immunogenicity and reactogenicity will reveal the molecular pathways underlying vaccine outcomes, and this therefore highlights the importance of large-scale GWASs in vaccinology.

Transcriptomic analyses have recently shown significant potential for studying responses to various vaccines, describing early (hours/days) gene signatures following vaccination that were highly predictive of subsequent measures of immunogenicity for seasonal influenza [6]. Our ability to accurately predict specific responses is a core utility of high-throughput methods in vaccine development, because early signatures could provide novel correlates of protection and reveal early mechanisms that are critical in the development of specific responses.

Recently, NGS approaches have been used to describe the B-cell receptor repertoire following vaccination; this could form a new measure of immunogenicity and be used to identify sequences for the generation of therapeutic monoclonal antibodies [7]. Moreover, coupling of NGS and mass-spectrometry proteomic analyses has been used to generate data to establish the specific B-cell repertoire patterns that are associated with serum IgG responses following tetanus toxoid vaccine [8].

Complementing transcriptomic data, high-resolution proteomics has provided longitudinal profiles of the protein expressed following trivalent inactivated influenza vaccine, a vaccine containing three inactivated influenza strains [9]. High-dimensional flow cytometry, and more recently mass cytometry, have been exploited to interrogate changes in the cellular compositions before and after vaccination [10]. Although proteomics and mass cytometry analyses are less developed than other methods, these technologies represent an unprecedented opportunity to understand the complex cellular interplay underlying the development of antibody and T-cell responses. The overarching goal of high-throughput methods is to generate sequential mechanistic understanding of the specific immune activation induced by the different vaccine components (antigen, carrier protein or adjuvant) that ultimately results in protection. Although characterizing these immunological ‘building blocks’ is a daunting task, it is clear that high-throughput technologies have already revolutionized vaccinology research. Taking advantage of these developments is likely to guide us to targeted stimulation of the immune system to achieve specific and functional responses.

## Implications for clinical trials

Many vaccine candidates never make it past the clinical trials stages of development. The reasons for this include safety concerns in early trials, lack of immunogenicity in human subjects, and finally the significant costs associated with efficacy trials. Systems biology approaches are likely to address some of these issues by generating more detailed knowledge, both to feed into vaccine design and in early-stage vaccine evaluation. Particularly, understanding the molecular biology underlying vaccine responses is the first step towards targeted vaccine or adjuvant development to yield optimal immune responses. Critical to this is the establishment of accurate sampling frameworks to adequately capture the presence and amplitude of the molecular events (often short-lived) that underlie immunological responses. Initially, this requires frequent sampling and integrative analyses of multifaceted datasets within hours and days of vaccination. High-throughput approaches may also provide the high-resolution depiction of responses to investigational vaccines needed to identify safety signals in early-phase vaccine development.

Clinical trials today are designed to measure a small number of markers in many participants, but high-throughput methods allow us to measure large numbers of markers in smaller cohorts. The vast amount of data collected using these approaches lends itself to modeling of molecular response signatures (genetic, transcriptional or cell sub-populations) that predict vaccine outcome, including immunogenicity, reactogenicity and efficacy. Once such models are sufficiently accurate and detailed, these data are likely to facilitate accelerated vaccine development at reduced cost.

## Bridging the gap between new technology and regulation

Despite the groundbreaking studies published in recent years, vaccine development and assessment still rely on measurement of traditional immunological endpoints, and systems biology has not become integral to most vaccine trials. Particularly new is the idea of using such data to support licensing decisions. Collecting large datasets and validating the use of them in carefully conducted proof-of-principle studies using efficacious vaccines with a known correlate of protection is the key for validating the use of high-throughput methods for the assessment of future vaccines. Once specific immunological patterns associated with protective vaccination outcome or markers of safety have been reliably established, immunological big data may become useful in supporting vaccine licensing and providing an important baseline for post-licensing monitoring of safety. Generating thoroughly validated data in addition to a close dialog with regulators is pivotal to making these approaches viable in the regulatory environment.

Access to high-throughput technologies at decreasing costs now provides the opportunity to unlock the mechanisms underlying vaccine-induced protection and generate critical knowledge to accelerate vaccine development in preparation for infectious disease threats in the future.
